# Systems analysis and improvement approach to improve naloxone distribution within syringe service programs: study protocol of a randomized controlled trial

**DOI:** 10.1186/s13012-023-01288-x

**Published:** 2023-08-03

**Authors:** Christopher F. Akiba, Sheila V. Patel, Lynn D. Wenger, Antonio Morgan-Lopez, Gary A. Zarkin, Stephen Orme, Peter J. Davidson, Alex H. Kral, Barrot H. Lambdin

**Affiliations:** 1 RTI International, 3040 E Cornwallis Rd, Research Triangle, Research Triangle Park, NC 27709 USA; 2 Department of Medicine, Division Global Public Health, UCSD, 9500 Gilman Dr, La Jolla, CA 92093 USA

**Keywords:** Systems Analysis and Improvement Approach, Opioid overdose deaths, Equitable naloxone distribution, Randomized controlled trial, Syringe service programs

## Abstract

**Background:**

More than half a million Americans died of an opioid-related overdose between 1999 and 2020, the majority occurring between 2015 and 2020. The opioid overdose mortality epidemic disproportionately impacts Black, Indigenous, and people of color (BIPOC): since 2015, overdose mortality rates have increased substantially more among Black (114%) and Latinx (97%) populations compared with White populations (32%). This is in part due to disparities in access to naloxone, an opioid antagonist that can effectively reverse opioid overdose to prevent death. Our recent pilot work determined that many barriers to naloxone access can be identified and addressed by syringe service programs (SSPs) using the Systems Analysis and Improvement Approach to Naloxone distribution (SAIA-Naloxone). This randomized controlled trial will test SAIA-Naloxone’s ability to improve naloxone distribution in general and among BIPOC specifically.

**Methods:**

We will conduct a trial with 32 SSPs across California, randomly assigning 16 to the SAIA-Naloxone arm and 16 to receive implementation as usual. SAIA-Naloxone is a multifaceted, multilevel implementation strategy through which trained facilitators work closely with SSPs to (1) assess organization-level barriers, (2) prioritize barriers for improvement, and (3) test solutions through iterative change cycles until achieving and sustaining improvements. SSPs receiving SAIA-Naloxone will work with a trained facilitator for a period of 12 months. We will test SAIA-Naloxone’s ability to improve SSPs’ naloxone distribution using an interrupted time series approach. Data collection will take place during a 3-month lead-in period, the 12-month active period, and for an additional 6 months afterward to determine whether impacts are sustained. We will use a structured approach to specify SAIA-Naloxone to ensure strategy activities are clearly defined and to assess SAIA-Naloxone fidelity to aid in interpreting study results. We will also assess the costs associated with SAIA-Naloxone and its cost-effectiveness.

**Discussion:**

This trial takes a novel approach to improving equitable distribution of naloxone amid the ongoing epidemic and associated racial disparities. If successful, SAIA-Naloxone represents an important organizational-level solution to the multifaceted and multilevel barriers to equitable naloxone distribution.

**Supplementary Information:**

The online version contains supplementary material available at 10.1186/s13012-023-01288-x.

Contributions to the Literature

•This study represents the first trial to assess the effectiveness of the Systems Analysis and Improvement Approach for Naloxone (SAIA-Naloxone) to improve distribution of the lifesaving medication.
•This trial also acknowledges and targets racial disparities within the opioid overdose epidemic as strategy activities focus on improving naloxone distribution to Black, Indigenous, and people of color (BIPOC).
•If successful, SAIA-Naloxone may provide an effective systems-level solution to an epidemic challenged by multilevel barriers.

## Reporting Standards

A standardized checklist aided this protocol’s clarity and transparency. The CONSORT checklist helped to clarify this protocol’s design, conduct, analysis, and interpretation.

## Background

More than half a million Americans died of an opioid-related overdose between 1999 and 2020, the majority of which occurred between 2015 and 2020 [1]. In recent years, the mortality crisis has been exacerbated by an increased flow of synthetic opioids into the drug supply and challenges related to the COVID-19 pandemic. In November 2021, the CDC reported that 100,306 opioid overdose deaths occurred in the 12 months prior, an increase of 28.5% from the year before [2]. Racial disparities are prevalent in these data, with opioid overdose mortality rates having increased disproportionally among Black (114%) and Latinx (97%) populations compared to White populations (32%) since 2015 [3]. Further, in Americans older than 55, opioid overdose mortality rates among non-Hispanic Black men are four times greater than the overall rates among others in the same age group [4].

Despite alarming increases, opioid overdose mortality is entirely preventable with the administration of naloxone, a medication that reverses the opioid-induced respiratory depression that leads to fatal overdose [5, 6]. However, access to naloxone remains limited, especially in populations with the most overdose deaths [7–9]. Numerous challenges result in insufficient and inequitable access to naloxone including systemic racism and inadequate screening, refill, and data systems [10–14]. Despite these challenges, our recent pilot work determined that many barriers can be mitigated when addressed by syringe service programs (SSPs). Nationally, 94% of SSPs provide naloxone [12] and research from California has shown that SSPs reach and provide key health services for people who are disproportionately affected by health disparities, including Black, Indigenous, and people of color (BIPOC) [15–18].

To improve naloxone distribution overall and address disparities in distribution throughout California, we designed a randomized controlled trial to test the ability of an implementation strategy (the Systems Analysis and Improvement Approach [SAIA]) to enhance naloxone distribution by SSPs and ultimately reduce overdose deaths and overdose mortality disparities. SAIA is a multifaceted implementation strategy that supports frontline service providers in gaining a comprehensive view of the cascade of services they provide, identifying areas for improvement, and iteratively testing new approaches to improve their delivery of the full cascade [19, 20]. The original SAIA trial improved services to prevent mother-to-child HIV transmission [21], with clinic staff describing the strategy as easy to use and practical [22]. Since then, SAIA has been adapted to improve hypertension, mental health, and adult and pediatric HIV care systems [23–26].

The present study responds to the opioid overdose mortality crisis and related disparities by determining the effectiveness of SAIA at improving naloxone distribution in general, and among BIPOC in particular, at SSPs throughout California. To that end, our study aims include.

1. testing the effectiveness of SAIA-Naloxone in improving naloxone distribution at SSPs,
2. testing the effectiveness of SAIA-Naloxone in improving naloxone distribution at SSPs to BIPOC and other key subgroups, and
3. estimating the cost and cost-effectiveness of SAIA-Naloxone on improving equitable access to naloxone at SSPs.

This trial will be one of the first to utilize SAIA (1) within the United States and (2) among community-based organizations, specifically SSPs, and the only to (3) impact naloxone distribution and to (4) target equity-centered outcomes. The trial is funded by the National Institute on Drug Abuse (grant #1R01DA055277-01).

## Methods/design

### Trial context and setting

Opioid overdose mortality has shifted in California in recent years from prescription opioids to heroin and fentanyl. From 2011 through 2017, mortality due to prescription overdose decreased 6% while overdose deaths due to heroin and fentanyl increased precipitously at 89% and 320%, respectively [27]. Ethnic disparities also persist within overdose deaths, with data from the California Opioid Overdose Surveillance Dashboard showing that Native Americans had significantly higher rates of prescription opioid and fentanyl overdose deaths compared to other groups [27]. The authors concluded that data-informed interventions to address opioid overdose deaths are required to address these disparities [27]. Regarding naloxone distribution in our own California-based research, we found that Black and Latinx PWID are 25% and 47% less likely, respectively, to receive naloxone compared to White PWID [28]. Our SAIA-Naloxone pilot data demonstrated that many barriers are surmountable and can be addressed by SSPs, leading to the present study [28].

SSPs provide access to and disposal of syringes and injection equipment for PWID and offer a variety of other prevention and treatment services. They are ideal organizational settings for naloxone distribution because they have staff who are culturally competent in providing services for PWID, and PWID already engage with and trust SSPs to care for their health [29]. By state law, only SSPs authorized by the California Department of Public Health (CDPH) may possess syringes and naloxone for distribution [28]. At the time of this publication, all California SSPs authorized by CDPH have adopted naloxone distribution. However, despite this, implementation of naloxone distribution must improve to reduce population-level mortality rates and address disparities.

### Naloxone delivery cascade

SSPs distribute naloxone to participants through a series of sequential steps that help ensure effective use. This typically beings with SSP staff routinely engaging participants in naloxone screening. Screening takes the form of staff-initiated discussion during service utilization to gauge participants’ awareness of naloxone and knowledge regarding its administration. If SSP staff determine that a participant would benefit from naloxone training and distribution and the participant signals interest, staff provide a brief training on effective naloxone administration. SSP staff then distribute initial doses or provide refills during participants’ subsequent visits. During each visit, staff also work with participants to determine their interest in secondary distribution so that others in their communities may benefit from their visit. There may be inefficiencies throughout this process that need to be addressed to improve naloxone distribution among PWID seeking services at SSPs.

### The Systems Analysis and Improvement Approach (SAIA)

SAIA is an implementation strategy that facilitates an organizational level analysis of service delivery by assigning a trained SAIA specialist to apply tools and techniques and engage staff to define barriers, identify solutions, and evaluate their success in cycles until achieving desired change [19]. This structured yet iterative process takes place over three main steps. The first step utilizes the *cascade analysis tool* (CAT), a visual aid that uses clinic data to help the specialist and staff identify participant drop-offs along the delivery cascade. Gimbel and colleagues (2016) identified the CAT as adaptable in their original SAIA trial, and subsequent SAIA studies have modified the tool to fit an array of cascades across varied health systems [19, 22–26, 28]. The second step focuses on *process mapping*, where the specialist assists staff in documenting the sequential steps of participant care to understand potential causes of participant drop-off. *Continuous quality improvement* (CQI) comprises the final step, which focuses on group design of “micro-interventions,” solutions intended to reduce drop-offs identified during the process mapping step. Once the team selects solutions to test, the staff will implement and later, alongside the specialist, evaluate their impact on targeted points of drop-off. They will then repeat this final step until they achieve the desired change [19]. The developers of SAIA consider these final two steps to be core components [22].

### SAIA-Naloxone

This trial tests an adaptation of SAIA in SSPs for the purpose of improving naloxone distribution. The three steps remain unchanged, although we have tailored them to the specific organizational setting and evidence-based intervention.

#### Naloxone cascade analysis tool (NCAT)

Together with the SAIA-Naloxone specialist, SSP staff will use the NCAT to assess their site’s naloxone delivery cascade and identify participant drop-offs in general and with BIPOC specifically [30]. SSP staff will collaborate with the SAIA-Naloxone specialist to identify areas they would like to prioritize for systems improvement based on the specifics of their SSP’s delivery cascade.

#### Process mapping

Alongside the specialist, SSP staff will draw process maps to document and visualize the flow of participants across their site’s naloxone delivery cascade. The SAIA-Naloxone specialist will then work with staff to review their service structure, discuss potential root causes of drop-offs, and identify solutions that might streamline workflow and reduce key points of drop-off. Finally, SSP staff will decide as a group which solutions to implement and what the planned roles and responsibilities will be for implementation.

#### Continuous quality improvement (CQI)

SSP staff will proceed in implementing the selected solutions in their SSP for at least 4 weeks. Some solutions might involve systems redesign, streamlining services, and/or improving knowledge and beliefs among SSP staff. For example, if SSP staff designed a solution focused on increasing naloxone trainings with BIPOC participants, the specialist might encourage staff to consider system redesign; linkage of SSP staff to trainings, webinars, and other resources that address aspects of racial equity and anti-Black racism in overdose prevention; and delivery of naloxone services by BIPOC staff. The specialist will help SSP staff identify the extent to which their solution improved components of the naloxone delivery cascade. Based on those results, SSP staff may choose to further adjust the current solution, design and test additional solutions in conjunction, or test different solutions entirely. Once SSP staff make a decision, the CQI cycle repeats. Allowing time for startup, SSPs are expected to complete up to eight CQI cycles during the 12-month active phase of the trial. Some SSPs may complete fewer cycles depending on the solutions they choose to implement and test, as certain solutions may require more time to implement or a greater time horizon to observe an impact.

### Specification and fidelity assessment

Specification of implementation strategies promotes component identification, measurement, and replicability through detailed explanation of a strategy’s actors, actions, action targets, temporality, dose, outcomes, and justification [31–33]. Despite its utility, strategy specification in published implementation literature is imprecise, contributing to a vague “black box” of implementation activities that often challenge the ability to understand and replicate strategies [34, 35]. Fidelity assessment of implementation strategies acts in conjunction with specification and facilitates an additional understanding of the extent to which strategies were implemented as intended [36]. Like specification, reports of implementation strategy fidelity are lacking and ultimately impede researchers’ abilities to interpret study findings [36, 37]. Perhaps most critically, fidelity assessment allows researchers to determine the likelihood of a type III research error, or failure to carry out a strategy as planned, leading to an erroneous conclusion that null results are due to attributes of the strategy itself, rather than to its poor application [38].

The present study takes a theory-informed approach to combine implementation strategy specification and fidelity assessment. Table 1 describes the SAIA-Naloxone implementation strategy in line with Proctor et al.’s (2013) specification recommendations [33].

**Table 1:** SAIA-Naloxone implementation strategy specification and fidelity

Name it		Systems Analysis and Improvement Approach for Naloxone (SAIA-Naloxone)
Define it		Facilitate development of a quality monitoring system to conduct cyclical small tests of change led by the organizational implementation teams
Specify it	Actor	External specialist works and facilitates discussion with the organization’s implementation team with regards to the SAIA-Naloxone process
Actions		1. Identify gaps: a. Present data evaluating the SSP’s naloxone delivery cascade with NCAT b. Facilitate discussions and support the implementation team to identify and develop consensus with regards to the areas of attrition along the naloxone cascade that they would like to address 2. Identify causes and opportunities: a. Facilitate discussions with the implementation team to review the SSP’s service structure and draw process maps documenting the flow of participants through the naloxone delivery cascade to understand (i) why there are drop-offs or inequities in distribution at different points (root causes of participant attrition) and (ii) what it would take to address those issues (opportunities to streamline workflows and address key points of attrition) b. Assist team in developing consensus about programmatic modifications based on their importance and feasibility 3. Conduct CQI: a. Support and mentor the implementation team in operationalizing programmatic modifications b. Present follow-up data on the naloxone delivery cascade for the implementation team to assess changes resulting from programmatic modifications c. Repeat above actions after conclusion of the cycle
Action target		Leverage programmatic data to facilitate CQI and foster a learning climate
Temporality		After training the implementation team on the SAIA process and integrating enhanced instruments to collect program data into workflows to track naloxone delivery cascade indicators
Fidelity		Content: Specialist completes actions 1–3 with SSPs Coverage: Relevant SSP staff are present during actions 1–3 Duration: Actions 1, 2, and 3 take ~ 60min each Frequency: Specialist visits SSPs in person during months 1, 4, 7, and 10. Specialist meets SSPs virtually during all other months in active phase Quality: Specialist forms rapport with SSP staff, flexibly attends to their unique needs, and motivates SSP staff to engaged with actions 1–3 Participant responsiveness: SSP staff like working with the specialist and like participating in actions 1–3
Targeted implementation outcomes		1. Implementation effectiveness relative to IAU a. Reach: Number of participants screened for naloxone engagement b. Fidelity (to the naloxone cascade): Number of participants who receive naloxone 2. Equitable implementation effectiveness relative to IAU a. Reach: Number of BIPOC and other key groups screened for naloxone engagement b. Fidelity (to the naloxone cascade): Number of BIPOC and other key group participants who receive naloxone
Justification		SAIA-Naloxone combines a broad view of the service system with iterative improvement cycles in a user-friendly way; by leveraging SAIA-Naloxone, SSPs can identify fillable gaps in the naloxone delivery cascade and apply locally generated solutions that have a higher likelihood of leading to measurable and sustained improvements in fidelity to the cascade and penetration of naloxone

*BIPOC* Black, Indigenous, and people of color, *IAU* implementation as usual, *CQI* continuous quality improvement, *NCAT* naloxone cascade analysis tool, *SSPs* syringe services programs

The authors’ original recommendations included identifying a strategy’s “dose,” or its frequency and intensity [33]. Given its similar conceptual alignment with fidelity, we expanded the dose categorization to include additional elements of traditional fidelity assessment. Using Carroll et al.’s (2007) conceptual framework for implementation fidelity, we bolstered the original “dose” category to include content, coverage, frequency, duration, quality of delivery, and participant responsiveness [39]. Integrating fidelity assessment within specification responds to calls from the implementation literature to improve reporting of both [34–37]. In doing so, we not only clarify SAIA-Naloxone activities but also outline expectations regarding their fidelity and how we plan to assess it.

### Trial aims and hypotheses

We plan to examine SAIA-Naloxone’s impact in SSPs compared to an implementation-as-usual (IAU) condition across three aims and several related hypotheses.

#### Aim 1

Our first aim is to test the effectiveness of SAIA-Naloxone on improving naloxone distribution within SSPs. We hypothesize that compared with SSPs receiving IAU, SSPs receiving SAIA-Naloxone will significantly increase the number of people receiving naloxone and number of naloxone doses distributed during the 12-month active period. Further, we hypothesize that SSPs receiving SAIA-Naloxone will significantly increase the number of people receiving naloxone in the 6 months after the active period (the sustainment period) compared with SSPs receiving IAU.

#### Aim 2

Our second aim will test the effectiveness of SAIA-Naloxone on improving naloxone distribution at SSPs to BIPOC and other key subgroups. We hypothesize that SAIA-Naloxone will significantly increase the number of BIPOC and women receiving naloxone from them during the 12 months active period and during the 6-month sustainment period. To test this hypothesis, we anticipate utilizing a subset of SSPs that report disaggregated outcome data based on participant level demographics like race, ethnicity, and gender.

#### Aim 3

Our third aim will estimate the cost and cost-effectiveness of SAIA-Naloxone on improving equitable access to naloxone at SSPs, relative to IAU. We hypothesize that, relative to IAU, SAIA-Naloxone will be cost-effective at increasing the number of people receiving naloxone from SSPs. We also hypothesize that, relative to IAU, SAIA-Naloxone will be cost-effective at increasing the number of BIPOC receiving naloxone from SSPs.

**Fig. 1:**
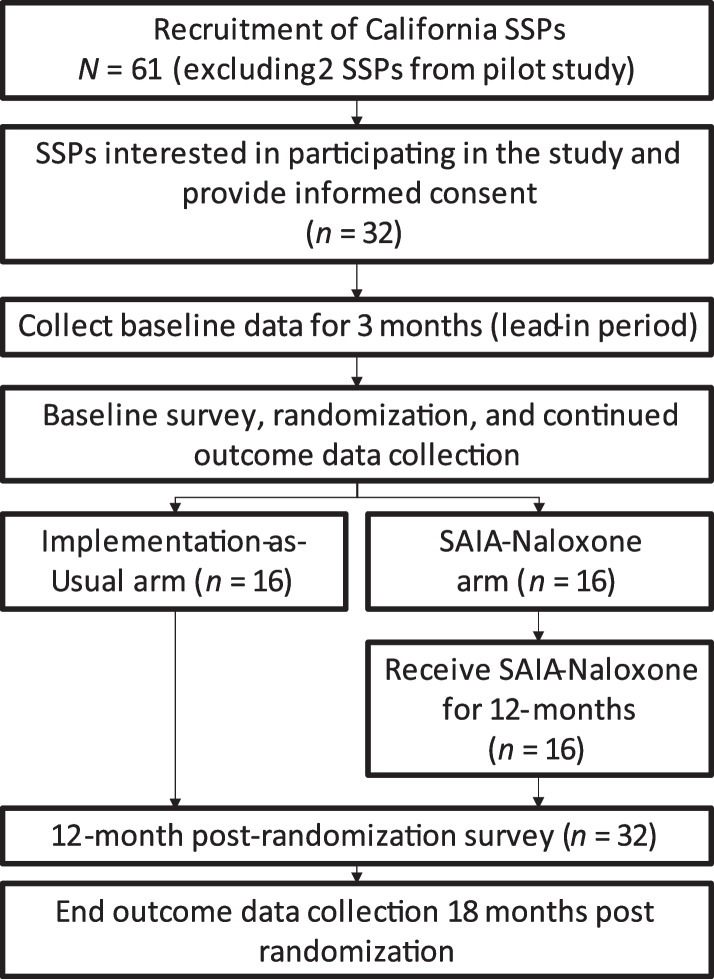
SAIA-Naloxone consort diagram

To evaluate these aims, we plan a randomized controlled interrupted time series trial with 32 SSPs in California. We will randomly assign 16 SSPs to the SAIA-Naloxone arm and 16 SSPs to IAU (Fig. 1). SSPs randomized to the IAU arm will not receive support to improve naloxone distribution. SSPs in California have already adopted naloxone distribution. We are therefore testing the ability of SAIA-Naloxone to optimize naloxone distribution within SSPs. Accordingly, we characterize IAU by the absence of SAIA-Naloxone with the goal of comparing whether SAIA-Naloxone improves SSPs’ Naloxone distribution. We will harmonize primary outcome data collection across SSPs receiving SAIA-Naloxone and IAU as described in the “Trial variables and data collection” section below.

### Pre-randomization and randomization procedures

The following eligibility criteria apply for study participation: SSP is located and operates in California; SSP is authorized by CDPH; and SSP has distributed naloxone to participants in the past 30 days. We will exclude SSPs who participated in our pilot study (*n* = 2) or do not distribute naloxone (currently *n* = 0) [28]. Prior to randomization, SSPs that enroll in the trial will enter a 3-month lead-in period to collect outcome data regarding the number of SSP participants, the number of people receiving naloxone, and the number of naloxone doses distributed overall, disaggregated by BIPOC and women (Table 2) [28]. With technical support from the study team, SSPs will collect these data by tablet and smart phone. The team will work with SSP staff to integrate the tool into their workflow so that the program can capture pre-randomization lead-in data and post-randomization outcome data with minimal service disruption.

**Table 2:** SPIRIT flowchart

	Study period				
	Enrollment	Lead-in	Randomization	Active phase	Sustainment phase
Time point	Month 0	Months 1–3	Month 3	Months 4–15	Months 16–21
Enrollment activities:					
Eligibility screen	X				
Consent	X				
Randomization			X		
Strategy activities:					
SAIA strategy activities				X	
Implementation as usual (IAU)				X	
Assessments:					
Primary outcome data collection		X		X	X
Contextual data collection			X	X	
Fidelity data collection				X	X
Cost and cost-effectiveness data collection				X	X

After the 3-month lead-in period, we will reach out to the SSPs’ organizational directors and arrange a call to conduct a baseline survey. Baseline surveys will focus on contextual data like SSPs’ internal and external characteristics detailed further in our “Trial variables and data collection” section. Our preliminary work determined that SSPs affiliated with public health departments possess vastly different service structures compared to non-health department affiliated SSPs [28]. Additionally, a recent analysis of SSP funding composition revealed that programs awarded with California Harm Reduction Initiative (CHRI) funds, a grant mechanism that provides SSPs with relatively less-restrictive and longer-term funding, provided more comprehensive services compared to SSPs without CHRI funding [40]. Therefore, we will stratify programs by health department affiliation and CHRI funding status. Within each stratum, the study coordinator will randomly allocate SSPs 1:1 to either study condition.

### SAIA-Naloxone specialist activities

Our study team will hire and train specialists with experience distributing naloxone in SSPs and who are familiar with SSP culture. The specialist training will leverage mock data to simulate the steps of SAIA-Naloxone and focus on electronic data collection instruments, the NCAT, racial disparities, anti-Black racism, and racial equity tools for overdose prevention. After training, the study team will provide weekly supervision to specialists throughout the 12-month active phase, with more intensive supervision during the first 2 months.

For SSPs in the experimental arm, specialists will partner with site leadership and frontline staff during the 12-month active phase. The specialist will meet with these individuals twice per month for the first 3 months and once per month for the remaining 9 months. Specialists will be allotted 45 h of time to deliver SAIA-Naloxone to each SSP (3 h per meeting: 1 h for the meeting itself and 2 additional hours for time spent preparing for or following up after the meeting), which aligns with the amount of time allotted for similar activities in the original SAIA trial [19]. At the first visit, the specialist will discuss the naloxone delivery cascade and SAIA-Naloxone in detail with SSP staff, observe the SSP’s process for distributing naloxone, and review data collection approaches. Having laid this groundwork, the specialist will guide the SSP through the three iterative steps of SAIA-Naloxone until achieving desired change.

### Trial variables and data collection

#### Implementation determinants and context variables

Our naloxone pilot study identified implementation climate and leadership engagement as important implementation determinants that can be influenced by SAIA-Naloxone and ultimately improve naloxone distribution among SSPs [28]. Therefore, in the present study, we will assess change in implementation climate and leadership engagement over time. We will first collect SSP-specific contextual data at randomization (baseline) and 12 months after randomization from all enrolled sites. We will ask the primary contact at each SSP about basic organizational characteristics (location, number of staff, budget, etc.). Next, we will ask the primary contacts as well as other staff involved with naloxone distribution at each SSP about contextual variables such as implementation climate and leadership engagement for improving naloxone distribution [41–43].

#### Fidelity variables

We will assess SAIA-Naloxone fidelity at the specialist level. Assessment will utilize descriptive statistics such as means/medians, standard deviations/interquartile ranges, and ranges given the small sample of specialists employed by the study (*n* = 2). Fidelity to SAIA-Naloxone focuses on assessing the domains of content, coverage, frequency, duration, quality, and participant responsiveness of SAIA-Naloxone. To monitor fidelity, all meetings between SAIA-Naloxone specialists and SSPs will be audio recorded, and the specialist will document each meeting with an SSP in a site-specific encounter log that includes the duration of the encounter, the roles of meeting attendees, and which of the three steps the specialist completed. Study staff will rate meeting content, quality, and participant responsiveness by reviewing 20% of recorded sessions using a fidelity checklist. To assess frequency, duration, and coverage, study staff will review and assess each encounter log.

#### Primary outcome variables

Primary outcomes center on the multidimensional variable of implementation effectiveness. Implementation effectiveness “is an organization-level construct that refers to the aggregated consistency, quality, and appropriateness of innovation use within an organization” [44]. Implementation effectiveness fits our broad objectives, focused on the extent to which SAIA-Naloxone impacts naloxone distribution at the organizational level. Accordingly, SSPs represent the primary unit of analysis. The Proctor et al. (2011) taxonomy of implementation outcomes forms our operationalization of implementation effectiveness, focusing on SSPs’ reach of naloxone screening and fidelity to naloxone cascade completion [45].

Aim 1 focuses on SAIA Naloxone’s ability to improve implementation effectiveness relative to IAU. We will assess “reach” as a proxy for the implementation effectiveness concepts of appropriateness and consistency, evaluating the number of participants screened for naloxone engagement, while accounting for the total number of participants who present for services. We will assess fidelity (i.e., fidelity to cascade completion) as a proxy for the implementation effectiveness concept of quality, evaluating the number of people who receive naloxone, again accounting for the total number of SSP participants screened for naloxone distribution. We collected similar indicators from SSPs in prior studies due to their utility in analyses and feasibility during collection [9, 10, 28]. Aim 2 focuses on SAIA-Naloxone’s ability to improve implementation effectiveness for BIPOC and other key participant subgroups relative to improvements in SSP participants overall. Therefore, operationalization of Aim 2 outcomes mirror those from Aim 1 but with a focus on those groups. Furthermore, our focus on the overall and disaggregated proportion of people receiving naloxone stems from a strong body of research showing that such outcomes significantly predict opioid overdose mortality rates at the SSP level [46–48].

We will harmonize primary outcome variable collection timing between SAIA-Naloxone and IAU SSPs, with both submitting data electronically on a fixed schedule. SSPs will receive prorated financial incentives for the number of completed data submissions at 6, 12, and 18 months after randomization. Based on our prior work, we anticipate retaining 98% of SSPs’ data submissions for 12 months after randomization and 92% of submissions at 18 months [28].

#### Cost and cost-effectiveness variables

We will collect cost outcomes using a modified version of the Substance Abuse Services Cost Analysis Program (SASCAP) [49], widely applied and adapted to a variety of behavioral and public health interventions [50–55]. The modified SASCAP will capture resource use and costs from the SSP provider perspective, as this perspective best aligns with SAIA-Naloxone’s focus on improving the naloxone delivery cascade as measured by the number of people receiving naloxone (including disaggregated numbers for BIPOC and other key subgroups) and the number of doses distributed. To collect the resources used for each of the three SAIA-Naloxone steps, the SASCAP will systematically track the time, space resources, and any material resources utilized by SAIA-Naloxone specialists and SSP staff involved in the strategy over the active and sustainment phases. We will also collect data on the time spent training SAIA-Naloxone specialists, SSP staff wages, building costs, and material costs.

### Data analysis and power calculation

Before model fitting, we will assess whether there is significant variation across the three potential levels of aggregation for each outcome: (1) within-SSP level (repeated measures over time), (2) between-SSP level, and (3) county level. We will also examine the functional form of changes over time in outcomes. We assume that piecewise linear (i.e., linear change during the 3-month lead-in period, post-implementation linear change from baseline through 18 months, and a period of treatment effect “deterioration” (if any) between 12 and 18 months) will be the predominant functional form, though with this large number of assessments, nonlinear forms may be necessary [56–58]. We will estimate a random intercept (*π*_*0is*_), which is the estimated (conditional) mean value of the outcome at time = 0 (e.g., lead-in baseline), and two random slopes (*π*_*1is,*_* π*_*2is*_): (1) the estimated per-year change in Y from the lead-in phase through 18 months; and (2) the “deterioration” phase, capturing how much reduction there is in change over the last year, if any, for the SAIA-Naloxone condition.

Level 2 will examine the between-SSP level effects. The key predictor included in *X*_*qis*_ is a 0/1 dummy indicator indicating whether SSP *i* was in the SAIA-Naloxone condition or IAU. *β*_*1s*_ will capture the average change over time in naloxone distribution (and other outcomes), which (1) should not be significantly different from zero during the lead-in period and (2) will vary by SAIA-Naloxone and IAU after implementation (by virtue of the SAIA-Naloxone × time period 1 interaction). *β*_*0s*_ will capture the conditional mean level of naloxone distribution, which, if it varies by implementation condition, would capture a mean shift in our study outcomes. *β*_*2s*_ will capture the change over time in naloxone distribution during the second year; if it interacts with the SAIA-Naloxone indicator, it will capture slope differences over time beyond 1 year between SAIA-Naloxone and the IAU condition, with Year 1 gains maintained if this parameter estimate is nonsignificant.

Level 3 will capture county-level variability in intercepts and, if necessary, slopes over time. As mentioned previously, our Aim 2 analysis will use the subset of SSPs that report outcome data disaggregated by race, ethnicity, and gender. For both Aims 1 and 2, we will reject the null hypothesis (i.e., there are no differences between SAIA-Naloxone and IAU SSPs regarding the test statistics described above) if *p*-values are equal to or less than an alpha coefficient of 0.05.

We structured a Monte Carlo power analysis [59] using the parameters from our pilot. For Aim 1, we structured a population model based on the number of weekly data submissions from SSPs) with a binary predictor structured to have the following effects, using the lead-in period standard deviation across 1 year in the pilot (15.09) as the denominator for the effect size. The mean shift in post-implementation outcomes was 22.76 additional people served under SAIA-Naloxone, yielding a Cohen’s *d* of 1.50. The per-week increase in number served under SAIA-Naloxone was 1.29, reaching a longitudinal Cohen’s *d* effect size [60, 61] of 1.0 by 11 weeks. We generated 250 synthetic samples of *N* = 32 and analyzed the samples in Mplus while imposing the random effect structure from pilot estimates and a county-level intraclass correlation of 0.05. Specific to Aims 1 hypotheses that pertain to the active 12-month phase, we will have at least 80% power to detect a post-implementation mean shift and differences in slopes over time. Specific to Aims 1 hypothesis that pertain to the sustainment phase, detecting treatment effect deterioration from 12 to 18 months for 80% power requires deterioration equivalent to a Cohen’s *d* of 0.48. The extremely large number of repeated measures offsets the sample size of 32 with regard to statistical power to detect slope differences [62].

We will calculate cost estimates for each of the three SAIA-Naloxone steps. We will multiply the quantity of a resource (e.g., labor) used by its price (e.g., wage) and then calculate the average cost for each step. Our cost-effectiveness analysis approach will follow the methods our authorship group have implemented in previous studies [50, 51, 53–55]. We will combine our cost estimates with the estimated changes in outcomes, the number of people receiving naloxone, and the number of BIPOC receiving naloxone. To derive cost-effectiveness ratios, we will calculate the difference in costs and outcomes between the two arms. We will then calculate the incremental cost-effectiveness ratio (ICER) as the ratio of the difference in costs to the difference in outcomes. For example, for effectiveness defined as the number of people receiving naloxone, the ICER represents the incremental cost spent for an additional person receiving naloxone. We will also calculate cost-effectiveness acceptability curves (CEACs) [63–65]. The CEACs incorporate the inherent joint variability of the cost and effectiveness estimates, and they show the probability that an intervention is cost-effective as a function of the policymaker’s intrinsic valuation or willingness to pay for the clinical outcome. We will use nonparametric bootstrap methods to calculate CEACs.

## Discussion

This trial represents several important advancements within implementation research. Since the initial SAIA trial to improve prevention services for mother-to-child HIV transmission in Kenya, Mozambique, and Côte d’Ivoire in 2014, the strategy has been adapted several times for the purposes of improving hypertension, mental health, and adult and pediatric HIV care systems across the region [23–26]. To our knowledge, this trial will be one of the first randomized trials of SAIA in the United States and among SSPs, and the first focused on improving the naloxone cascade and on equity-centered outcomes.

The study’s U.S. focus comes at a critical moment, as the country has experienced the highest levels of opioid overdose mortality in its history [2, 3, 66]. Racial disparities are present within the epidemic with BIPOC experiencing some of the least favorable outcomes [3, 4]. In response to the crisis, lawmakers at the federal level and in every state have enacted some type of naloxone access legislation to improve distribution of the lifesaving treatment [67–71]. Despite these legal efforts, naloxone distribution remains insufficient against the backdrop of persistently increasing opioid-related fatalities [5]. To combat the epidemic and the disparities within, researchers have called for multifaceted strategies focused on community-based organizations that address major barriers—such as structural racism—to improve naloxone distribution [30, 72–74]. SAIA-Naloxone represents an attentive response to these calls given its focus on working within SSPs and toward equity-focused outcomes. SSPs are often community-based programs that PWID trust to provide quality health care amidst systems that criminalize and stigmatize substance use [29, 75]. SAIA-Naloxone’s multifaceted and multilevel strategies also respond to the complex and unique needs of different SSPs throughout California. If successful, SSPs may utilize recently released federal and state funds focused on curbing the epidemic to adopt SAIA-Naloxone as a means of improving naloxone distribution.

This trial also takes a pragmatic and theory-informed approach to specifying SAIA-Naloxone and assessing its fidelity. Our approach combines Proctor et al.’s (2017) strategy specification recommendations with Carroll et al.’s (2007) conceptual framework of implementation fidelity [33, 39]. In doing so, we hope to clearly report the strategy’s activities as well as assess the extent to which they were carried out as intended. Combining structured specification and fidelity assessment might improve our understanding of not only how the strategy works but also of the likelihood that impacts on outcomes are due to the strategy itself and not to other factors (i.e., assessment of a type III research error). Fidelity results may additionally highlight facets of the strategy’s implementation that future applications could harness or improve.

The current study builds upon existing evidence for the SAIA strategy [23–26] as well as our own pilot work that brought SAIA into SSPs [28]. If successful, SAIA-Naloxone may represent a pragmatic and scalable systems approach to combating the opioid overdose mortality crisis and the disparities within.

### Supplementary Information

**Additional file 1. **Consort checklist

## Data Availability

N/A.

## Abbreviations

BIPOC: Black, Indigenous, and people of color
CAT: Cascade analysis tool
CDPH: California Department of Public Health
CEAC: Cost-effectiveness acceptability curve
CHRI: California Harm Reduction Initiative
CQI: Continuous quality improvement
IAU: Implementation as usual
ICER: Incremental cost-effectiveness ratio
NCAT: Naloxone cascade analysis tool
PWID: People who inject drugs
SAIA: Systems Analysis and Improve Approach
SASCAP: Substance Abuse Services Cost Analysis Program
SSP: Syringe service program
